# Are monoclonal antibodies effective in patients with severe obesity in SARS‐CoV‐2 infected?

**DOI:** 10.1002/iid3.771

**Published:** 2023-02-09

**Authors:** Claudio Ucciferri, Livia Moffa, Samanta Moffa, Jacopo Vecchiet, Katia Falasca

**Affiliations:** ^1^ Department of Medicine and Science of Aging, Clinic of Infectious Diseases University “G. D'Annunzio” Chieti Pescara Italy; ^2^ Department of Pharmacy University “G. D'Annunzio” Chieti Pescara Italy

**Keywords:** COVID‐19, monoclonal antibody, obesity

## Abstract

It is important to block SARS‐CoV‐2 infection immediately with early therapies, such as monoclonal antibodies (MonoAbs). Also, several studies show that obesity is associated with a high risk of severe COVID‐19 disease. We enrolled 32 SARS‐CoV‐2 infected patients who received MonoAbs, all patients were not vaccinated for SARS‐CoV‐2, and they received therapy after 7 ± 2 days from the onset of COVID‐19 symptoms. In the days following administration, patients followed home therapy with Pidotimod 800 mg bid for 10 days and cholecalciferol 2000 UI for 20 days, prescribed the same day they received MonoAbs therapy. Our study found that there are no differences in the therapeutic response between obese and nonobese patients with SARS‐CoV‐2 infection undergoing MonoAbs therapy, in fact, none of them underwent hospitalization. Furthermore, the effect of the immunostimulant Pidotimod and cholecalciferol may have contributed to the resolution of COVID‐19 symptoms in these patients.

## Graphical abstract

There are no differences in the therapeutic response between obese and nonobese patients with SARS‐CoV‐2 infection undergoing MonoAbs therapy The effect of the immunostimulant Pidotimod and cholecalciferol may contribute to the resolution of COVID‐19 symptoms in patients with SARS‐CoV‐2 infection. Early therapies in COVID‐19, such as monoclonal antibodies, reduce the risk of progression to severe disease.

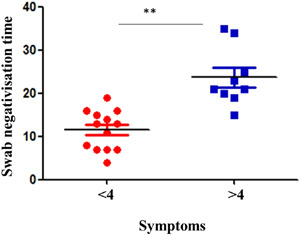

## 1 INTRODUCTION

The coronavirus disease 2019 (COVID‐19) pandemic, due to the SARS‐CoV‐2 virus, has changed everyone's life, causing about 14 million deaths in the world.^1^

Symptoms and signs of SARS‐CoV‐2 infection are not always specific and range from fever, dry cough, fatigue, headache, dysgeusia, anosmia, and acute lung injury with shortness of breath to acute respiratory distress syndrome, which can lead to patient death.^2^

Many factors are associated with severe outcomes from COVID‐19, and obesity is one of these. In fact, patients with a body mass index (BMI) above 30 kg/m^2^ are at high risk for progressing to severe COVID‐19, hospitalization, and/or death. A high BMI is associated with a reduction in residual volume, functional status, and responsiveness of the respiratory system. Furthermore, obese patients infected with SARS‐CoV‐2 may have a higher mortality rate related to obesity‐related elevated inflammatory cytokines.^3^ Indeed, the levels of particular proteins such as chemokines or cytokines are increased in the plasma of patients with obesity due to the secretion of cytokines with inflammatory activity (interleukin‐10, 1 and 6 and tumor necrosis factor‐alpha) from adipocytes.^4^

Recently, several meta‐analyses showed that obesity is associated with a poor prognosis of COVID‐19 because it causes chronic inflammation, increases angiotensin‐converting enzyme 2 (ACE2) expression facilitating the cellular entry of SARS‐CoV‐2.^5^, ^6^

Moreover, these patients are at risk because they often have other associated diseases and comorbidities such as hypertension, type 2 diabetes, dyslipidemia, and cardiovascular disease.^7^ In addition, chronic inflammation, characterized by high levels of cytokines, alters the innate immune system, and SARS‐CoV‐2 infection can aggravate this condition.^5^, ^8^, ^9^

Therefore, in these patients is important to block SARS‐CoV‐2 infection soon as possible with early therapies, such as monoclonal antibodies (MonoAbs).

The aim of this study was to verify if the therapeutic response to the administration of MonoAbs in patients with severe obesity (BMI ≥ 35 kg/m^2^) was the same as that in patients with normal BMI (between 20 and 25 kg/m^2^). Considering there is a standard dosage for MonoAbs, it could be thought that in obese patients, the efficacy of the therapy is worse considering the different volumes of distribution.

The secondary outcome of this study was to highlight differences in the time of negativization between the two groups of patients, understood as the time elapsed between the administration of the MonoAbs and the first negative molecular rhinooropharyngeal swab for SARS‐CoV‐2.

## 2 METHODS

We enrolled 32 SARS‐CoV‐2 infected patients who received MonoAbs from April 2021 to May 2021 in the Unit of Infectious Diseases at the “Policlinico SS. Annunziata of Chieti, Italy” (Table 1).

**Table 1:** Characteristics and clinical symptoms of COVID‐19 patients

	Nonobese patients (BMI [kg/m^2^] = 23.47 ± 1.4)	Obese patients (BMI [kg/m^2^] = 38.07 ± 3.7)
Mean age (in years)	73.20	52.55
Sex (male)	40%	50%
Sex (female)	60%	50%
*Symptoms*		
Cough/sore throat	70%	77.27%
Fever	60%	72.73%
Fatigue	50%	50%
Muscle ache/myalgia	60%	54.55%
Diarrhea	30%	27.27%
Burning skin	0%	4.54%
Dysgeusia	0%	13.64%
Anosmia	10%	13.64%
Ageusia	10%	13.64%
Headache	30%	22.73%
Dyspnea	20%	22.73%

Abbreviation: BMI, body mass index.

All patients were not vaccinated for SARS‐CoV‐2, and they received therapy after 7 ±  2 days from the onset of COVID‐19 symptoms.

In the days following administration, patients followed home therapy with Pidotimod 800 mg bid for 10 days and cholecalciferol 2000 UI for 20 days, prescribed the same day they received MonoAbs therapy, casirivimab, and imdevimab by Roche.

The study was approved by the local institutional review board of G. d'Annunzio University, Chieti‐Pescara in May 2022 (No. 03‐18/05/2022), and all patients provided written informed consent for the use of MonoAbs.

Of the 32 SARS‐CoV‐2 infected patients enrolled, 22 were obese (BMI = 38.07 ± 3.7 kg/m²), and 10 nonobese (BMI = 23.47 ± 1.4 kg/m²). Obese patients had an average age of 52.55 ± 15.34. The nonobese patients had a mean age of 73.2 ± 9.

All patients underwent blood chemistry tests (CBC, PCR, PCT, creatinine, eGFR, ferritin, liver, and lipid profile), blood gas analysis, and a questionnaire about the symptoms of COVID‐19 on the day of the administration of the MonoAbs. Blood chemistry samples were repeated after 3 months.

The patients were contacted by telephone after the administration of MonoAbs to check if there had been an improvement in COVID‐19 symptoms after treatment and to check if anyone had undergone reinfection with SARS‐CoV‐2.

### 2.1 Statistical section

Statistical analysis tests were assessed using Prism Statistical software version 5.0 (GraphPad Software, Inc.). We compared data from the two groups of patients (obese and nonobese) using t test and Mann–Whitney test. A value of *p* < .05 was considered statistically significant.

## 3 RESULTS

The results of our study demonstrate that none of the patients in both groups (obese and nonobese) infected with SARS‐CoV‐2 who received MonoAbs were hospitalized and the time to negativization of obese patients (16 ± 8 days) is less than nonobese patients (20 ± 8 days), with the same time elapsed between the onset of symptoms and the administration of MonoAbs (7 ± 2 days).

This data could be affected by the difference in age between the two groups under examination (nonobese patients are older).

**Figure 1:**
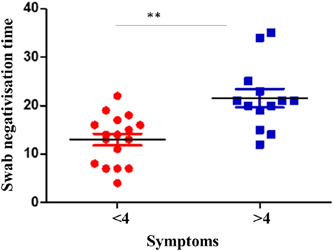
There is significance between swab negativization time and total symptoms if only obese patients are considered

**Figure 2:**
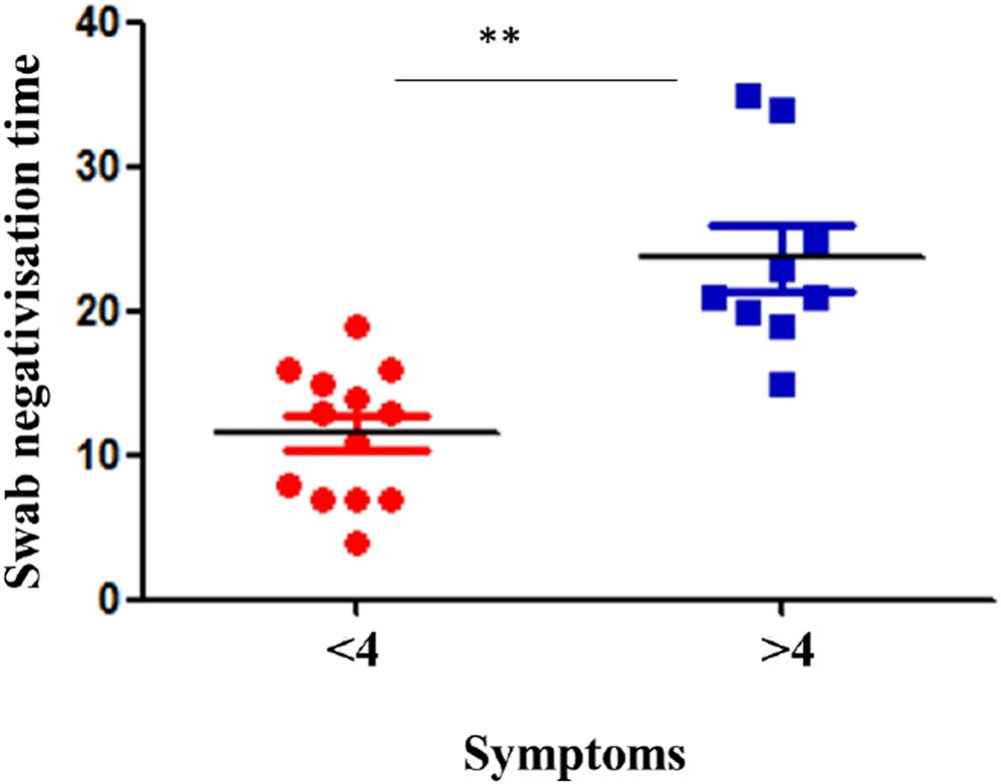
There is significance between swab negativization time and the total number of symptoms (*p*‐value < .001). Patients with symptoms less than 4 negativized first.

Analyzing the data, it also emerged that there is significance between the time of negativization and total symptoms (*p*‐value < .001) only in obese patients and not if we consider all patients in a single group (shown in Figure 1). Obese patients with less than 4 symptoms were negativized earlier (shown in Figure 2).

This difference between the two groups is most likely due to the fact that the population under consideration is small and that it was difficult to find unvaccinated patients undergoing therapy in the selected period.

**Figure 3:**
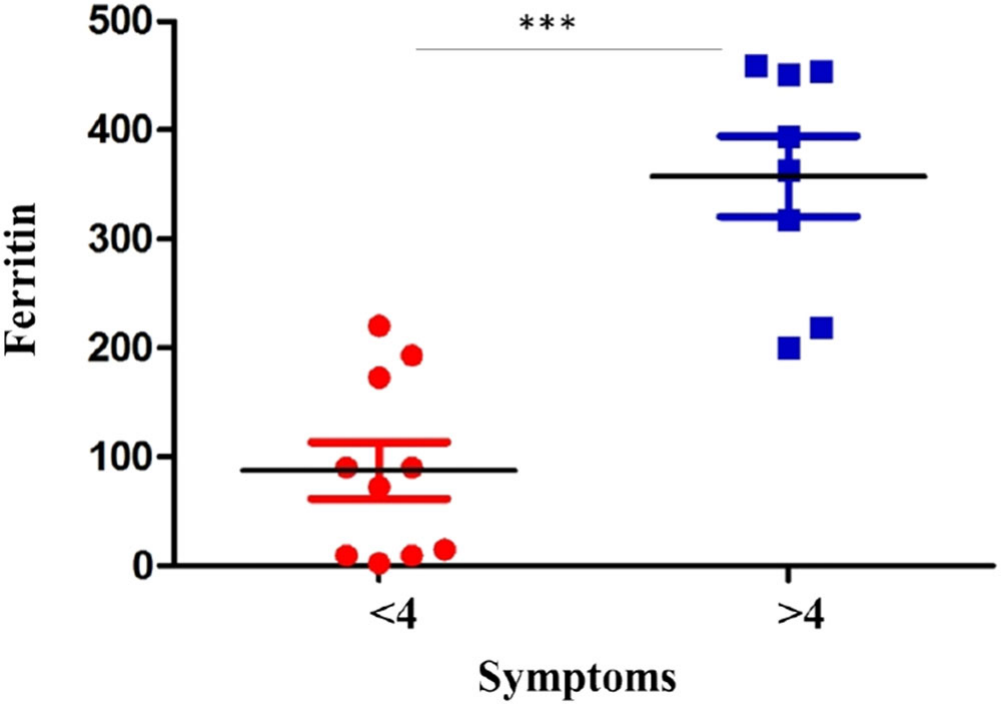
Symptoms greater than 4 are associated with higher ferritin levels.

Another significant finding that emerged is the correlation between ferritin and the number of symptoms. Those who had more COVID‐19 symptoms at baseline showed higher ferritin levels (*p* = .0002 and *r* = .75; shown in Figure 3).

Three months after MonoAbs therapy, the patients were contacted by telephone and subjected to a questionnaire relating to the symptoms of COVID‐19. It was found that 68.1% of obese patients and 60% of nonobese patients reported improvement in asthenia and myalgia within the first 2 days of MonoAbs administration. In addition, blood tests performed 3 months after MonoAbs therapy revealed a worsening of the lipid profile with an increase in total and LDL cholesterol, most likely due to the sedentary life of the months of post‐covid rather than to the administration of MonoAbs.

## 4 DISCUSSION

In this study, we demonstrate that there are no differences in hospitalization between obese and nonobese SARS‐CoV‐2 positive patients who received MonoAbs therapy.

Thirty‐two patients were enrolled in our study, and none of them underwent hospitalization or progressed to severe COVID‐19 disease, regardless of BMI. This means that MonoAbs therapy is the same effective in obese and nonobese patients.

To the best of our knowledge, there are no studies in the literature that indicate equal MonoAbs efficacy in terms of hospitalization rates in obese and nonobese patients. This is the first study that proves it. In fact, MonoAbs are administered with the same dosage in obese and nonobese patients, but in obese patients, the volume of distribution is higher.

In fact, many studies linked the severity of COVID‐19 to obesity. It is a condition characterized by chronic inflammation of various organs. SARS‐CoV‐2 infection aggravates this process. There are various mechanisms that link obesity and the severity of COVID‐19. Therefore, understanding that MonoAbs are as effective as the rest of the population in these patients is very important. Moreover, the patients in our study are also unvaccinated, and we expected they could get worse, but they did not have a serious illness.

Another surprising data is enlistment time. MonoAbs were administered more than 7 days after the onset of symptoms. Recent studies suggest that efficacy is reduced when administered for more than 7 days.^10^

For example, according to a retrospective case‐control study, patients with COVID‐19 who received MonoAbs within the first 5 days of symptom onset experienced no disease progression compared with those who received MonoAbs after 6 days.^11^, ^12^

But in our study, despite the timing of administration, the efficacy of the therapy was confirmed in obese and nonobese patients. This finding could be explained by the administration of home therapy with pidotimod and cholecalciferol.^13^

The immunomodulant Pidotimod and cholecalciferol may improve the immune system response against SARS‐CoV‐2.^10^ Pidotimod decreases IL‐6 production, while cholecalciferol can regulate immune function during COVID‐19.^14^, ^15^ The double working, on viruses (MonoAbs) and on the immune system to avoid cytokine storm (pidotimod and cholecalciferol), could be the explanation why these patients did not get worse.

Patients received MonoAbs with a time of more than 7 days from the beginning of the symptoms with consequently reduced efficacy of the therapy.^12^ Furthermore, obese patients, particularly those at risk of suffering from severe COVID‐19 disease, had complete resolution of symptoms within a few days of MonoAbs administration. Our attention is focused more on obese patients because they are more at risk of developing SARS‐CoV‐2 pneumonia and hospitalization in intensive care.^16^

From our study, another interesting data point emerged. Obese patients with fewer symptoms were negativized earlier, establishing a significant correlation between symptoms and time of negativization only in this group. This difference between the two groups could be explained by the fact that the sample under examination is small and that the group of nonobese patients is made up of older patients.

In addition, obese patients were negativized earlier than nonobese patients despite there were no differences in the timing of administration of MonoAbs. These data are in contrast with recent literature because studies correlate comorbidities and also obesity to a long‐lasting risk of positivity for SARS‐CoV‐2.

But the studies also correlate age to a longer positive time for COVID‐19.^17^ From what emerges from our study, it could mean that advanced age is a stronger factor for long‐term positivity than obesity.

This should be better investigated with future studies.

One last fact that emerged from the study was that obese patients with more symptoms negativized late and had elevated ferritin levels on blood tests. Many studies linked high ferritin levels with fatal outcomes of COVID‐19.^18^ Furthermore, in obese patients, considering the chronic inflammatory state, there are already high levels of ferritin regardless of the infection.^19^

So we demonstrated that MonoAbs remain a viable therapeutic option to treat COVID‐19 disease and its complications, especially in obese individuals. Although they were not vaccinated and had a very high risk of serious illness, the infection after therapy resolved without complications.

Our study found that there are no differences in the therapeutic response between obese and nonobese patients with SARS‐CoV‐2 infection undergoing MonoAbs therapy, in fact, none of them underwent hospitalization. Furthermore, the effect of the immunomodulant Pidotimod and cholecalciferol may have contributed to the resolution of COVID‐19 symptoms in these patients, also considering that the MonoAbs were administered later than the current indications of efficacy of the therapy in the prevention of the severe COVID‐19 disease and that selected patients, who had elevated ferritin levels, had a high risk for progressing to severe disease.

The limitation of our study is mainly due to the small sample. The strict temporal enrollment criteria limited the number of enrolled patients. in our opinion, this was necessary to prevent the emergence of new viral variants from confounding the study results. Furthermore, our patients are all unvaccinated. Today the vaccination campaign is very extensive, so it is difficult to find a large sample. This is the first report on MonoAbs efficacy in obese patients, so the results of this study should be investigated in future large studies

## AUTHOR CONTRIBUTIONS

*Conceptualization*: Claudio Ucciferri and Katia Falasca. *Methodology*: Jacopo Vecchiet. *Formal analysis*: Katia Falasca. *Data curation*: Claudio Ucciferri and Livia Moffa. *Writing – original draft*: Claudio Ucciferri and Samanta Moffa. *Writing – review and editing*: Claudio Ucciferri and Katia Falasca. All authors have read and agreed to the published version of the manuscript.

## CONFLICT OF INTEREST STATEMENT

The authors declare no conflict of interest.

## ETHICS STATEMENT

The study was conducted according to the guidelines of the Declaration of Helsinki and was approved by the local institutional review board of G. d'Annunzio University, Chieti‐Pescara, in May 2022 (No. 03‐18/05/2022), and all patients provided written informed consent for the use of MonoAbs.

## Data Availability

The data that support the findings of this study are available from the corresponding author upon reasonable request.
